# Discordance in gender role attitudes between spouses and its relationship with the risk biomarkers of cardiovascular diseases: a couple-level analysis

**DOI:** 10.1038/s41598-026-41697-8

**Published:** 2026-03-03

**Authors:** Kiho Sung, Junsol Kim, Yeong-Ran Park, Hyeon Chang Kim, Yoosik Youm

**Affiliations:** 1 Department of Sociology, Yonsei University, 50 Yonsei-ro, Seodaemun-gu, Seoul, South Korea; 2 Department of Sociology, University of Chicago, 1126 East 59th Street, Chicago, IL USA; 3 Department of Senior Business, Gangnam University, Yongin, Gyeonggi South Korea; 4 College of Medicine, Yonsei University, 50-1 Yonsei-ro, Seodaemun-gu, Seoul, South Korea

**Keywords:** Discordance, Gender role attitude, Cardiovascular risk, Risk biomarkers, Actor-partner interdependence model, Biomarkers, Cardiology, Diseases, Health care, Medical research, Risk factors

## Abstract

Cardiovascular disease (CVD) is prevalent among older adults, but studies often focus on individual spouses rather than couple dynamics. This study examines how interplay between spouses—specifically discordance in gender role attitudes—relates to CVD risk biomarkers, using couple-matched data from the Korean Social Life, Health, and Aging Project (KSHAP) on 308 older married couples (616 individuals). Actor-partner interdependence models (APIM) reveal that discordant gender role attitudes are significantly associated with higher CVD risk markers—higher systolic blood pressure, higher triglycerides (TG), lower high-density lipoprotein cholesterol (HDL-C), and a higher TG: HDL-C ratio—among wives only. These associations persist after adjusting for individual-level risk factors, including marital quality, sociodemographics, health behaviors, and depressive symptoms. However, no significant association is found with high-sensitivity C-reactive protein. Two groups of wives are identified as being at the greatest risk for CVD: traditional wives with egalitarian husbands and egalitarian wives with traditional husbands.

**Supplementary Information:**

The online version contains supplementary material available at 10.1038/s41598-026-41697-8.

## Introduction

Cardiovascular disease (CVD) is the leading cause of death worldwide^1^. In the United States, for instance, over 695,000 individuals died from heart disease in 2021, accounting for 20% of all deaths^2^. Similarly, in South Korea, CVD has become a primary cause of death, with mortality rates increasing significantly with age^3^. This study examines marital relationships as a major social risk factor for CVD, focusing specifically on the interaction between spouses’ gender role attitudes rather than solely on each spouse’s individual appraisal of the marriage.

Marriage has been shown to offer protective effects against CVD. For example, married individuals tend to live longer, experience better mental health^4,5^, and have a lower likelihood of developing cardiovascular disease^6^. A growing body of research also demonstrates an association between perceived marital quality and various cardiovascular risks. For instance, women with high marital satisfaction or positive marital quality exhibit lower CVD risk, as measured by biomarkers (e.g., blood pressure, cholesterol, inflammation) or four-year survival rates after congestive heart failure^7–9^. Conversely, higher marital strain, lower spousal support, and reduced marital satisfaction have been linked to abnormal cardiovascular reactivity, hypertension, and elevated inflammation markers^9–13^. While these studies have advanced our understanding of the link between marriage and CVD, most have focused on intrapersonal processes within marriage, overlooking its inherently interpersonal and interdependent nature^14^. One exception, Wang and Li^15^, highlights the importance of inter-spousal relationships for mental health, finding that women face “double jeopardy” when they have low job autonomy and their husbands hold traditional gender ideologies regarding household labor. This suggests that wives’ mental health is influenced not only by their work situation but also by their husbands’ gender role attitudes.

Gender-relational theory provides a useful framework for couple-level health research because it conceptualizes gender not simply as an individual attribute but as a set of social relations that are produced and reproduced through everyday interactions and institutional arrangements, with implications for health^16^. Consistent with this approach, gender and health research emphasizes that health patterns are shaped by gendered divisions of labor and power and by relational dynamics within intimate heterosexual partnerships, rather than by individual attitudes alone^17^. Public health scholarship therefore argues that making gender theory explicit helps move beyond descriptive sex differences by clarifying how gendered social arrangements generate patterned health risks^18^. In social contexts where patriarchal gender relations are long-established and strongly institutionalized, these arrangements may differentially allocate responsibilities and authority within marriage^16,17^. Consequently, discordance in spouses’ role expectations may be experienced as relational strain that is not necessarily symmetric for women and men. Gender medicine research similarly underscores that gender‑related factors—such as depression and household-related stress—disproportionately burden women and can impair cardiovascular outcomes even when biological differences might be favorable^19^. Together, these perspectives motivate examining couple-level discordance in gender ideology as a sociocultural exposure relevant to CVD risk.

Building on this framework, the present study examines whether discordance in spouses’ gender role attitudes is associated with CVD risk biomarkers, and whether the association differs for wives and husbands. Gender role attitudes are multidimensional, encompassing beliefs about the division of paid work and domestic labor as well as norms surrounding childcare, caregiving, household decision-making, and public participation. In this study, we focus on couples’ concordance versus discordance in the division-of-labor dimension, a central organizing axis of gendered expectations within marriage^20^. As spouses’ gender role attitudes continually shape each other’s roles and identities, discordant attitudes may create ongoing tension or conflict, adversely affecting mental and physical health. Indeed, such discordance has been associated with lower marital satisfaction and higher marital strain^21–23^. Additionally, attitudinal dissimilarity in marriage can induce negative emotions, interpersonal stress, and even depressive symptoms^24,25^. Studies indicate that the impact of marital quality on cardiovascular health is generally more pronounced among women than men^9,10^.

Our major research questions are as follows: How is discordance in spouses’ gender role attitudes related to CVD risk markers among older couples? And how does this relationship vary by gender? We use a community-based sample of older married couples (*N* = 616 individuals or 308 couples) from Korean rural villages, assessing each spouse’s gender role attitudes and six CVD risk biomarkers.

## Methods

### Data

We use a couple-matched, cross-sectional community sample from the Korean Social Life, Health, and Aging Project (KSHAP)^26–28^. The data were collected in two rural Korean villages, K and L, located on Ganghwa Island, South Korea. We aimed to enroll all older adults (aged 60 or older in village K and aged 65 or older in village L) and their spouses residing in these villages. These communities represent typical rural Korean settings where farming is the primary industry: approximately 70% of respondents are currently working, with 88% actively engaged in farming, according to our survey results.

From February 2014 to March 2017, face-to-face interviews were conducted at participants’ homes or local community centers—village K between 2014 and 2016, and village L between 2016 and 2017. Interviewees were also invited to participate in clinical assessments conducted either at the public health center or in their homes. During data collection, 1,750 residents were identified as eligible participants: 707 from village K and 1,043 from village L. Of these, 1,538 residents participated in the interviews, with 591 from village K and 947 from village L, achieving response rates of 83.6% for village K and 90.8% for village L. Additionally, 1,189 individuals underwent biometric assessments, comprising 445 from village K and 744 from village L. Our final sample includes 308 married couples (104 from village K and 204 from village L), totaling 616 participants, who had no missing data in the variables used for this study.

The study was approved by the Institutional Review Board of Yonsei University and conducted in accordance with relevant guidelines and regulations. All participants provided written informed consent (IRB numbers: YUIRB-2011-012-01, 7001988-202304-SB-152-06, 7001988-202111-HRBR-244-05, 7001988-202308-SB-307-05).

### Measures

#### Assessment of cardiovascular health

We measure three categories of CVD biomarkers. First, we assess systolic blood pressure (SBP) and diastolic blood pressure (DBP), which are associated with increased risks of hypertensive heart disease, stroke, heart attack, and heart failure^29^. Second, we use triglycerides (TG), high-density lipoprotein cholesterol (HDL-C), and the triglyceride to high-density lipoprotein cholesterol ratio (TG: HDL-C ratio), which is considered a strong predictor of CVD compared to other lipid markers, such as low-density lipoprotein cholesterol^30^. Both blood pressure and lipid markers are physiological biomarkers that directly reflect physical states related to CVD risk. Third, we measure high-sensitivity C-reactive protein (hsCRP), a functional indicator of systemic inflammation and a predictor of CVD^31^.

During the clinical assessment, resting SBP and DBP were measured three times using the oscilloscopic method with an automatic sphygmomanometer (Carescape Dinamap V100; GE Healthcare, Milwaukee, WI, USA). Before measurement, participants rested in a seated position for over five minutes, with cuffs applied to both the left and right upper arms. We first calculated the average of the three measurements from each arm. Then, we calculated the overall mean blood pressure by averaging these two arm-specific means. Blood samples were drawn from the antecubital vein of each participant. hsCRP, HDL-C, and TG were analyzed using enzymatic methods (ADVIA 1800, Siemens, Tarrytown, NY, USA) at a central research laboratory. Participants with hsCRP levels exceeding 10 mg/L were excluded due to potential acute infection or injury-induced inflammation^31,32^. The TG: HDL-C ratio was calculated by dividing TG by HDL-C. To address skewness, we log-transformed hsCRP, TG, HDL-C, and the TG: HDL-C ratio^32^. Notably, analyses without log transformation yielded similar results.

#### Measurement of gender role attitude

Gender role attitudes are multidimensional, but in this study we focus on one core domain—attitudes toward the gendered division of paid work and household labor—which is widely used in cross-national research and commonly represented along a traditional–egalitarian spectrum^33^. Individuals with traditional attitudes support a division of labor in which wives are responsible for unpaid housework while husbands focus on paid work. In contrast, those with egalitarian attitudes support similar roles for both wives and husbands^34^.

During the face-to-face interview, gender role attitude was assessed using a 5-point Likert scale item from the 2002 International Social Survey Program module “Family and Changing Gender Roles III” ^35^. Participants were asked to indicate their agreement or disagreement with the following statement: “A man’s job is to earn money; a woman’s job is to look after the home and family.” This statement is widely used across countries to assess gender role attitudes concerning the division of household labor^36^. Responses in the original survey range from 1 (strongly agree), 2 (agree), 3 (neither agree nor disagree), 4 (disagree), to 5 (strongly disagree), with higher scores indicating a more egalitarian gender role attitude in this study. Although this item is widely used in cross-national surveys to capture the division-of-labor dimension of gender ideology, it does not represent all domains of gender role attitudes. Moreover, because it is a single-item measure, we cannot assess scale reliability (e.g., internal consistency) as would be possible with multi-item instruments; accordingly, our measure should be interpreted as a domain-specific indicator rather than a comprehensive assessment of gender ideology.

#### Other covariates

We control for four categories of traditional risk factors: sociodemographic covariates, health behaviors, depressive symptoms, and perceived marital quality. First, for sociodemographic covariates, we include age (in years) and education (in years). Annual household income is categorized into four groups: under KRW 10 million (approximately USD 7,600), KRW 10–20 million (USD 7,600–15,200), KRW 20 million or higher (over USD 15,200), and missing. We also include working status and the village of residence (either village K or L).

Second, for health behaviors and medication use, we include drinking status (currently not drinking, currently drinking), smoking status (currently not smoking, currently smoking), and body mass index (BMI), categorized as normal or underweight (BMI < 23 kg/m²), overweight (23 kg/m² ≤ BMI < 25 kg/m²), and obesity (BMI ≥ 25 kg/m²). Additionally, we control for medication use for hypertension and hyperlipidemia.

Third, depressive symptoms are measured using the 20-item Center for Epidemiological Studies Depression (CES-D) Scale^37^. Participants were asked how often in the past week they experienced the following: 0 = less than once a week, 1 = 1–2 days a week, 2 = 3–4 days a week, and 3 = 5 days a week or more. Sample items include: (1) “I was bothered by things that usually don’t bother me”; (2) “I did not feel like eating; my appetite was poor”; (3) “I felt that I could not shake off the blues even with help from my family or friends”; (4) “I had trouble keeping my mind on what I was doing”; (5) “I was happy”; (6) “I felt depressed”; (7) “I felt that everything I did was an effort”; (8) “I felt hopeless about the future”; (9) “I thought my life had been a failure”; (10) “I felt I was just as good as other people”; (11) “My sleep was restless”; (12) “I felt fearful”; (13) “I talked less than usual”; (14) “I felt lonely”; (15) “I enjoyed life”; (16) “People were unfriendly”; (17) “I had crying spells”; (18) “I felt sad”; (19) “I felt that people disliked me”; and (20) “I could not get going.” Responses are recoded and summed to create a total score, with higher scores indicating more depressive symptoms.

Fourth, for perceived marital quality, we assess both positive and negative dimensions, as suggested by previous research^9,32^. Marital quality is measured using six questions: (1) “How close do you feel is your relationship with your spouse?” (1 = not very close or somewhat close, 2 = very close, 3 = extremely close); (2) “Do you and your spouse spend free time together or apart?” (1 = mostly apart, 2 = some together and some apart, 3 = mostly together); (3) “How often can you open up to your spouse?”; (4) “How often can you rely on your spouse?”; (5) “How often does your spouse make too many demands on you?”; and (6) “How often does your spouse criticize you?” (1 = never, hardly ever, or rarely, 2 = some of the time, 3 = often for questions 3 to 6). We conducted an exploratory factor analysis using the principal factor method. Consistent with prior studies^9^, our analysis suggests that these items form two dimensions: positive marital quality (items 1 to 4) and negative marital quality (items 5 and 6), as shown in Table S1. We create two factor scores for positive and negative marital quality by calculating the average scores.

### Statistical analysis

**Fig. 1:**
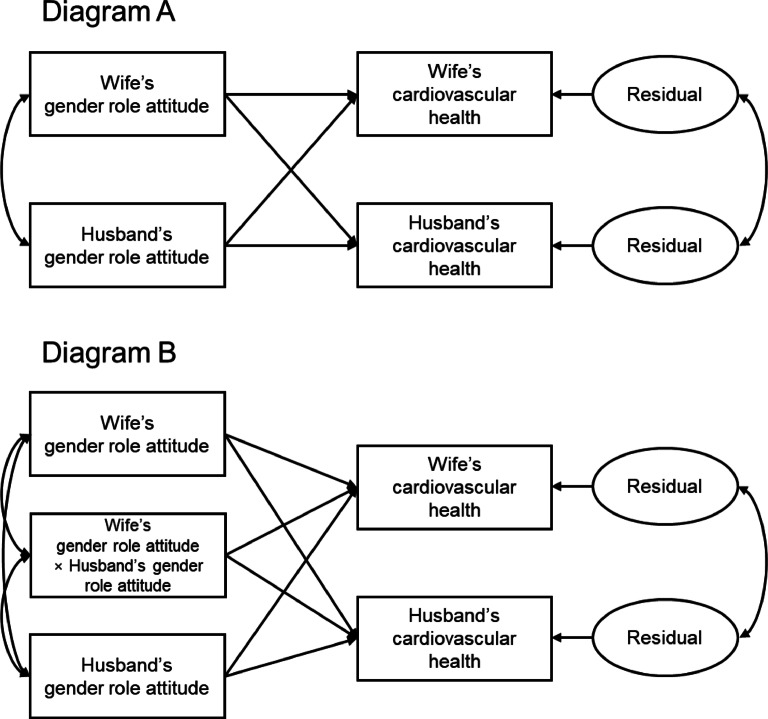
Conceptual framework of actor-partner interdependence model (APIM). Single-headed arrows refer to predictive paths, and double-headed arrows refer to correlated variables.

We estimate an actor-partner interdependence model (APIM) to examine the associations between discordance in spouses’ gender role attitudes and risk markers of CVD by gender, as shown in Fig. 1. The main distinction between ordinary least squares (OLS) regression and APIM is that APIM accounts for couple-level correlations in the residuals of dependent variables between spouses^38–41^. Married couples, particularly older couples who have shared decades of life together, are likely to have correlated CVD risk factors, including lifestyle behaviors (diet, physical activity, sleep) and shared family stress. Additionally, the residuals of all dependent variables are positively correlated between spouses, supporting the validity of APIM in this study. The Breusch-Pagan test of independence for each statistical model indicates that the residuals for some dependent variables, such as DBP, TG, and the TG: HDL-C ratio, are significantly correlated at conventional levels (*p* < .05), while residual correlations for SBP, hsCRP, and HDL-C are weaker. We apply APIMs to all dependent variables because the primary aim of this study is to examine the interdependence of gender role attitudes within couples and their impact in CVD risk biomarkers.

The APIM analyses proceed in two steps. First, we estimate APIMs including wives’ and husbands’ gender role attitudes (Fig. 1, Diagram A). Second, we add an interaction term between wives’ and husbands’ gender role attitudes (Fig. 1, Diagram B). If discordance in gender role attitudes between spouses is significantly associated with CVD risk markers, the interaction effect should be significant. In each of these steps, we first control for age, as CVD risk biomarkers generally increase with age, and subsequently adjust for all other covariates outlined before. The APIM coefficients can be interpreted similarly to OLS regression coefficients. APIMs are estimated using the seemingly unrelated regression (sureg) command in Stata 18.

## Results

### Participant characteristics

Table 1 presents the descriptive statistics for all variables included in the analyses. Overall, there are no statistically significant differences in CVD risk markers between women and men, except for DBP. Women tend to hold statistically significantly more egalitarian gender role attitudes than men, suggesting that many egalitarian wives have traditional husbands in our sample (see Table S2). The average age was 71.70 for women and 75.77 for men. The average years of education were relatively low, at 6.08 years for women and 8.33 years for men, reflecting the educational background of the older population in rural Korean villages.

**Table 1:** Descriptive statistics of analytical sample (*N* = 616 individuals/308 couples).

Variables	Women (*N* = 308)	Men (*N* = 308)	Gender difference (*p*-value)
	Mean (SD) or %	Mean (SD) or %	
SBP (mmHg)	137.86 (14.78)	139.24 (15.43)	0.127
DBP (mmHg)	78.55 (8.02)	80.83 (8.41)	< 0.001
hsCRP (mg/L) ^a^	1.46 (1.44)	1.31 (1.61)	0.027
TG (mg/dL)	145.43 (80.83)	145.25 (73.74)	0.964
HDL-C (mg/dL)	51.45 (12.24)	50.19 (12.96)	0.205
TG: HDL-C ratio	3.13 (2.21)	3.20 (2.07)	0.340
Gender role attitude (5 = very egalitarian)	3.14 (1.25)	2.79 (1.21)	< 0.001
Age (years)	71.70 (6.88)	75.77 (6.30)	< 0.001
Education (years)	6.08 (3.62)	8.33 (3.79)	< 0.001
*Annual household income*			0.795
Less than KRW 10 M (USD 7,600)	25.65%	25.97%	
KRW 10 M ~ 20 M (USD 7,600–15,200)	24.35%	24.68%	
KRW 20 M (USD 15,200) or higher	46.75%	47.40%	
Missing	3.25%	1.95%	
Currently working	78.90%	80.52%	0.616
Village L	66.23%	66.23%	1.000
Currently drinking	3.25%	42.86%	< 0.001
Currently smoking	0.65%	17.53%	< 0.001
*BMI (kg/m* ^*2*^ *)*			< 0.001
Normal or underweight (BMI < 23)	24.35%	39.29%	
Overweight (23 ≤ BMI < 25)	23.70%	24.35%	
Obesity (BMI ≥ 25)	51.95%	36.36%	
Medication for hypertension	52.60%	39.29%	0.001
Medication for hyperlipidemia	17.86%	10.71%	0.011
Depressive symptoms	6.14 (7.12)	4.60 (6.57)	0.002
Positive marital quality	2.65 (0.40)	2.70 (0.35)	0.077
Negative marital quality	1.15 (0.33)	1.12 (0.30)	0.247

T tests or Chi-square tests were performed to examine the gender differences between the means or the proportions. Wilcoxon signed-rank test was performed to examine gender difference between the median of hsCRP, TG, and TG: HDL-C ratio. SBP = systolic blood pressure; DBP = diastolic blood pressure; hsCRP = high-sensitivity C-reactive protein; TG = triglyceride; HDL-C = high-density lipoprotein cholesterol; BMI = body mass index. ^a^Respondents with hsCRP > 10 mg/L were excluded (*n* = 19 couples).

As shown in Table S3, the correlation coefficients between wives’ and husbands’ CVD risk markers are statistically significant for DBP and TG, indicating the need for statistical models that account for non-independence in spouses’ cardiovascular health, such as APIM^36^.

### The discordance in spouses’ attitudes and its association with CVD risk biomarkers

Table 2 presents the associations between discordance in spouses’ gender role attitudes and cardiovascular risks among wives. In Models 1a and 2a, we estimate models that include both the wife’s and husband’s gender role attitudes, while in Models 1b and 2b, we add an interaction term between the wife’s and husband’s gender role attitudes.

**Table 2:** Discordance of spouses’ gender role attitudes and cardiovascular risks in women. In Model 2a and 2b, we control for age, education, household income, working status, township, drinking, smoking, obesity, medication uses for hypertension and hyperlipidemia, depressive symptoms, and positive and negative marital quality.

	Model 1a (Controlled for age)		Model 1b (Controlled for age)		Model 2a (Controlled for all covariates)		Model 2b (Controlled for all covariates)	
	Coefficient	95% CI	Coefficient	95% CI	Coefficient	95% CI	Coefficient	95% CI
DV: SBP								
Wife attitude	− 0.43	− 1.69, 0.83	1.99	− 0.97, 4.95	0.57	0.73, 1.87	2.84+	0.05, 5.73
Husband attitude	− 0.39	− 1.68, 0.91	2.38	− 0.95, 5.71	− 0.13	1.41, 1.14	2.46	0.75, 5.67
Wife × Husband attitude			− 0.89+	− 1.89, 0.10			− 0.84+	1.80, 0.12
Observation	308							
DV: DBP								
Wife attitude	0.11	0.59, 0.81	− 0.11	1.76, 1.54	0.22	− 0.53, 0.97	− 0.11	− 1.78, 1.56
Husband attitude	0.24	0.48, 0.96	− 0.01	1.87, 1.85	0.10	− 0.63, 0.83	− 0.27	− 2.12, 1.59
Wife × Husband attitude			0.08	0.47, 0.63			0.12	− 0.43, 0.67
Observation	308							
DV: log(hsCRP)								
Wife attitude	− 0.08+	0.17, 0.01	− 0.01	0.23, 0.20	− 0.10*	− 0.20, − 0.00	− 0.01	0.23, 0.20
Husband attitude	0.06	0.04, 0.15	0.13	0.11, 0.37	0.04	− 0.06, 0.13	0.14	0.10, 0.37
Wife × Husband attitude			− 0.02	0.09, 0.05			− 0.03	0.10, 0.04
Observation	289							
DV: log(TG)								
Wife attitude	0.00	0.04, 0.04	0.12*	0.02, 0.21	0.01	0.04, 0.05	0.13**	0.04, 0.22
Husband attitude	− 0.03	0.07, 0.01	0.10+	− 0.00, 0.21	− 0.02	0.06, 0.02	0.12*	0.02, 0.22
Wife × Husband attitude			− 0.04**	− 0.08, − 0.01			− 0.05**	− 0.08, − 0.02
Observation	308							
DV: log(HDL-C)								
Wife attitude	− 0.01	0.03, 0.01	− 0.05*	− 0.10, − 0.00	− 0.02	0.04, 0.01	− 0.07**	− 0.11, − 0.02
Husband attitude	0.00	0.03, 0.02	− 0.05+	− 0.11, 0.01	− 0.01	0.03, 0.01	− 0.06*	− 0.12, − 0.01
Wife × Husband attitude			0.01+	− 0.00, 0.03			0.02*	0.00, 0.03
Observation	308							
DV: log(TG: HDL-C ratio)								
Wife attitude	0.01	0.04, 0.06	0.17**	0.05, 0.29	0.02	0.03, 0.08	0.20**	0.08, 0.32
Husband attitude	− 0.03	0.08, 0.03	0.15*	0.02, 0.29	− 0.02	0.07, 0.04	0.18**	0.05, 0.32
Wife × Husband attitude			− 0.06**	− 0.10, − 0.02			− 0.06**	− 0.10, − 0.02
Observation	308							

DV = dependent variable; CI = confidence interval; SBP = systolic blood pressure; DBP = diastolic blood pressure; hsCRP = high-sensitivity C-reactive protein; TG = triglyceride; HDL-C = high-density lipoprotein cholesterol. +*p* < .10. *p < .05. **p < .01. ***p < .001 (for two-tailed tests).

The results indicate that discordance in spouses’ gender role attitudes is statistically significantly associated with SBP, TG, HDL-C, and the TG: HDL-C ratio in wives. Notably, as shown in Models 1a and 2a, none of these markers—SBP, TG, HDL-C, and the TG: HDL-C ratio—are associated with either the wife’s or husband’s individual gender role attitudes. Only discordance between spouses is related to these cardiovascular risk markers.

**Fig. 2:**
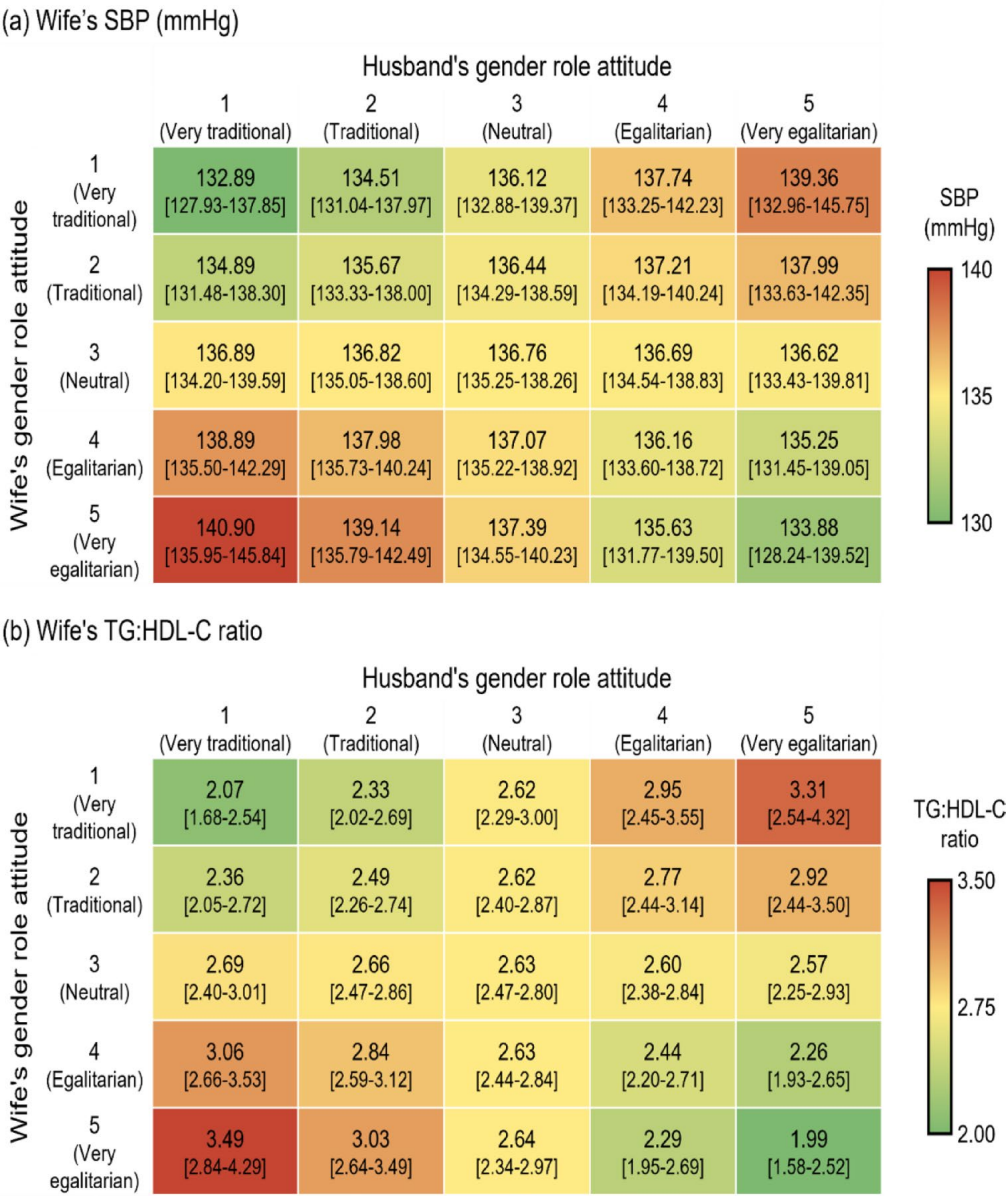
Heatmaps of wife’s risk biomarkers of cardiovascular disease predicted by interplay of spouses’ gender role attitudes. Numbers in the brackets indicate 95% confidence interval. SBP = systolic blood pressure; TG = triglyceride; HDL-C = high-density lipoprotein cholesterol.

**Fig. 3:**
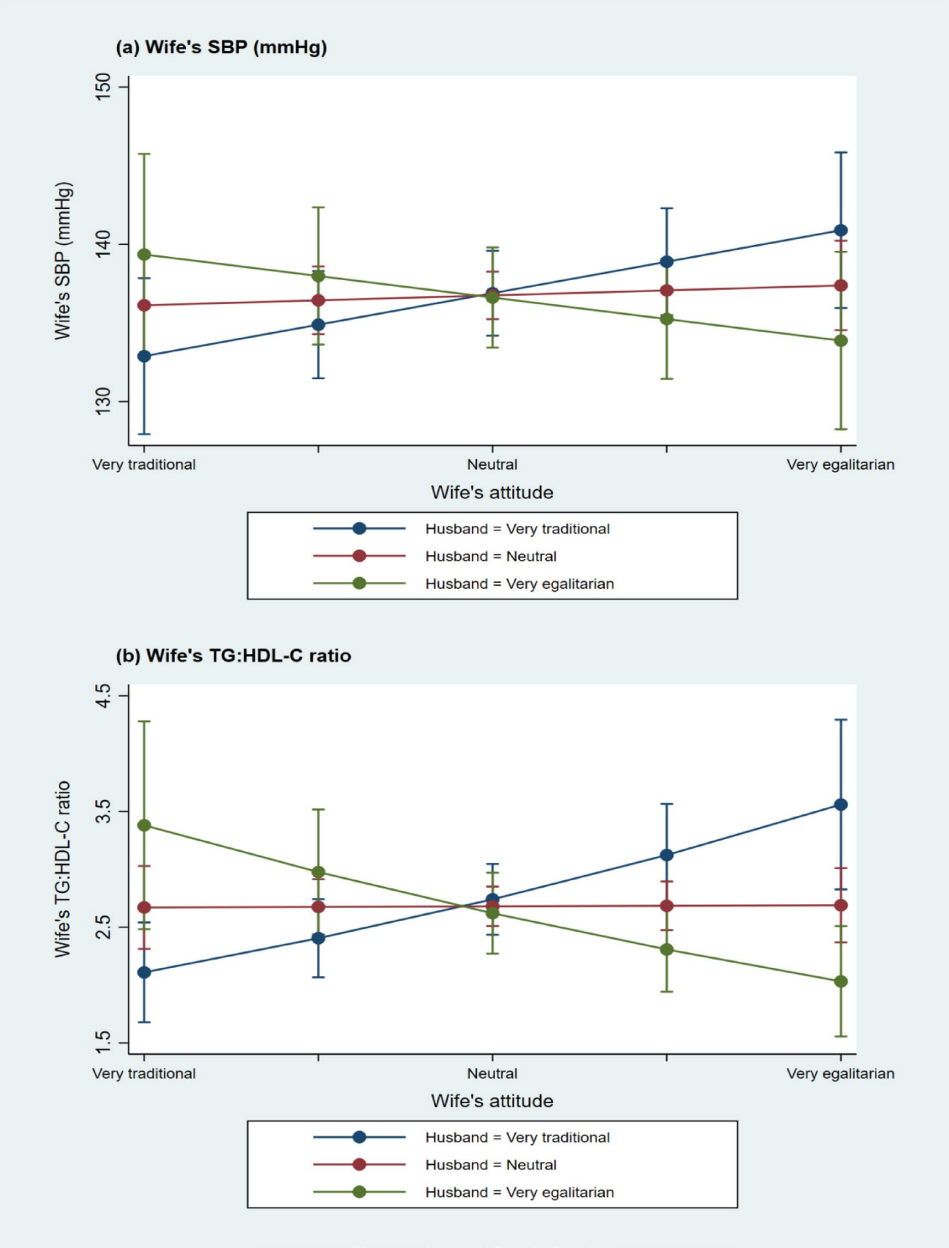
Discordance between spouses’ gender role attitudes and wife’s SBP and TG: HDL-C ratio. Bars in the panels indicate 95% confidence interval. TG = triglyceride; HDL-C = high-density lipoprotein cholesterol.

The findings for wives are illustrated in Figs. 2 and 3. These figures clearly demonstrate that it is the discordance in gender role attitudes between spouses—rather than the individual attitudes of either spouse—that is associated with higher cardiovascular risks for wives. In general, greater dissimilarity in spouses’ gender role attitudes correlates with elevated cardiovascular risk biomarkers in wives, regardless of each spouse’s individual stance.

In Fig. 2a, the husband’s attitude is represented in rows, while the wife’s attitude is shown in columns. The highest SBP levels for wives occur in two scenarios where the spouses’ attitudes are at their most discordant: when a very egalitarian wife has a very traditional husband, or when a very traditional wife has a very egalitarian husband. In contrast, the lowest SBP levels are observed when there is no attitudinal discordance—specifically, when both spouses are either very traditional or very egalitarian.

This pattern suggests that what primarily influences SBP is not each spouse’s individual attitude but the degree of discordance between them. The greater the discordance, the higher the wife’s average SBP. Moreover, the association between discordance and SBP intensifies as the spouses’ attitudes become more extreme. Consequently, lower SBP values are observed along the right diagonal of the matrix, where the spouses’ attitudes are identical; the lowest SBP values appear at the two ends of this diagonal. Conversely, higher SBP values are observed along the left diagonal, where attitudinal discordance is maximized, with the highest SBP values occurring at the two ends of this diagonal. The only exception occurs when either the wife’s or husband’s attitude is neutral; in such cases, the wife’s SBP remains low regardless of her spouse’s attitude.

Similar patterns are observed in Fig. 3 for the TG: HDL-C ratio. Figure 3 illustrates the predicted values and confidence intervals for SBP and the TG: HDL-C ratio in a format that allows easier reading of confidence intervals. However, no interaction effect of discordance is found for hsCRP. Only the wife’s gender role attitude is associated with her hsCRP level: the more egalitarian the wife’s attitude, the lower her hsCRP, implying reduced cardiovascular risk. Furthermore, no statistically significant association is observed between discordance and DBP.

The relationship between the husband’s CVD biomarkers and attitudinal discordance is summarized in Table 3. Unlike the findings for wives, no statistically significant associations are observed for husbands. The wife’s gender role attitude, rather than the interaction term between the spouses’ gender role attitudes, shows a marginally significant association with TG and the TG: HDL-C ratio for husbands. This suggests that attitudinal discordance between spouses is systematically related only to wives’ CVD biomarkers, not to husbands’.

**Table 3:** Discordance of spouses’ gender role attitudes and cardiovascular risks in men. In Model 2a and 2b, we control for age, education, household income, working status, township, drinking, smoking, obesity, medication uses for hypertension and hyperlipidemia, depressive symptoms, and positive and negative marital quality. DV = dependent variable; CI = confidence interval; SBP = systolic blood pressure; DBP = diastolic blood pressure; hsCRP = high-sensitivity C-reactive protein; TG = triglyceride; HDL-C = high-density lipoprotein cholesterol.

	Model 1a (Controlled for age)		Model 1b (Controlled for age)		Model 2a (Controlled for all covariates)		Model 2b (Controlled for all covariates)	
	Coefficient	95% CI	Coefficient	95% CI	Coefficient	95% CI	Coefficient	95% CI
DV: SBP								
Wife attitude	0.50	− 0.83, 1.82	0.83	− 2.33, 3.98	0.76	− 0.60, 2.12	0.84	− 2.24, 3.91
Husband attitude	− 0.86	− 2.24, 0.51	− 0.49	− 4.03, 3.05	− 0.71	− 2.10, 0.68	− 0.62	− 4.06, 2.81
Wife × Husband attitude			− 0.12	− 1.18, 0.93			− 0.03	− 1.05, 0.99
Observation	308							
DV: DBP								
Wife attitude	0.00	0.73, 0.72	0.05	1.68, 1.77	− 0.58	1.31, 0.15	− 0.47	2.12, 1.19
Husband attitude	0.26	0.49, 1.00	0.31	1.62, 2.24	0.01	0.74, 0.75	0.14	1.71, 1.98
Wife × Husband attitude			− 0.02	0.59, 0.56			− 0.04	0.59, 0.51
Observation	308							
DV: log(hsCRP)								
Wife attitude	0.02	− 0.07, 0.10	0.11	− 0.09, 0.31	0.01	− 0.08, 0.10	0.09	− 0.11, 0.29
Husband attitude	− 0.01	− 0.10, 0.07	0.09	− 0.14, 0.31	0.00	− 0.09, 0.09	0.10	− 0.13, 0.32
Wife × Husband attitude			− 0.03	− 0.10, 0.03			− 0.03	− 0.10, 0.04
Observation	289							
DV: log(TG)								
Wife attitude	− 0.03	− 0.07, 0.01	0.02	− 0.07, 0.11	− 0.04*	− 0.08, − 0.00	0.02	− 0.08, 0.11
Husband attitude	− 0.02	− 0.06, 0.02	0.04	− 0.07, 0.14	− 0.01	− 0.06, 0.03	0.05	− 0.05, 0.16
Wife × Husband attitude			− 0.02	− 0.05, 0.01			− 0.02	− 0.05, 0.01
Observation	308							
DV: log(HDL-C)								
Wife attitude	0.00	0.03, 0.02	0.01	0.04, 0.07	0.00	0.02, 0.03	0.02	0.03, 0.08
Husband attitude	0.00	0.03, 0.02	0.02	0.04, 0.08	0.00	0.03, 0.02	0.02	0.04, 0.08
Wife × Husband attitude			− 0.01	0.02, 0.01			− 0.01	0.02, 0.01
Observation	308							
DV: log(TG: HDL-C ratio)								
Wife attitude	− 0.03	0.08, 0.03	0.00	0.12, 0.13	− 0.05+	0.10, 0.01	− 0.01	0.13, 0.11
Husband attitude	− 0.01	0.07, 0.04	0.02	0.12, 0.16	− 0.01	0.07, 0.04	0.03	0.10, 0.17
Wife × Husband attitude			− 0.01	0.05, 0.03			− 0.01	0.06, 0.03
Observation	308							

+*p* < .10. *p < .05. **p < .01. ***p < .001 (for two-tailed tests).

## Discussion

A growing body of research has established a link between either a husband’s or wife’s perceived marital quality and cardiovascular health. However, relatively few studies have explored how the dynamics between spouses influence cardiovascular risks beyond each spouse’s individual behaviors or perceptions. To our knowledge, this study is the first to examine the role of discordance in spouses’ gender role attitudes in relation to cardiovascular risks, using a couple-matched community sample. We find that, in general, an individual spouse’s gender role attitude has limited influence, whereas the discordance itself plays a significant role: both egalitarian wives with traditional husbands and traditional wives with egalitarian husbands exhibit the highest CVD risk biomarkers, including elevated SBP, TG, and TG: HDL-C ratios and lower HDL-C. Notably, this association between discordance and CVD risk biomarkers is present only among wives, not husbands.

Interpreted through gender-relational approaches, discordance in spouses’ gender ideology can be understood not only as attitudinal mismatch but as a marker of strain in the gendered organization of everyday married life—i.e., how responsibilities, authority, and expectations are negotiated and enacted. In this perspective, discordance is not necessarily experienced symmetrically by women and men in settings where gender relations are long-established and institutionalized^16,17^. In older rural Korean communities, where the gendered division of labor has been settled over the life course, discordance may translate into greater day-to-day role strain for wives because they are more likely to remain accountable for domestic labor and care work regardless of personal ideology, whereas husbands may be comparatively buffered by decision authority and customary role entitlements. As a result, the practical consequences of discordance may more often contradict wives’ preferences than husbands’. For instance, egalitarian wives with traditional husbands may still bear the primary responsibility for household chores, contrary to their own egalitarian beliefs, while their husbands perform little housework in alignment with their traditional views. A similar dynamic applies to traditional wives with egalitarian husbands: such husbands may contribute minimally to farming or other financial responsibilities, as they may not perceive themselves as the primary breadwinner, a view that directly opposes the expectations of their traditional wives. These dynamics may be reinforced in later-life rural settings where cohort-linked marital expectations, limited formal supports for care, and livelihood arrangements (e.g., farming households) can concentrate routine domestic and caregiving responsibilities among women, amplifying women’s exposure to discordance-related strain. This interpretation also helps explain the absence of significant associations among husbands in our analyses, insofar as discordance may not substantially increase men’s day-to-day role demands under long-established patriarchal arrangements.

Several well-known studies support this interpretation. For example, Ross^42^ demonstrated using a national U.S. sample that the division of household labor is primarily shaped by the husband’s educational level and gender-role beliefs, with the wife’s education and attitudes having minimal influence. In another study, Greenstein^43^, using data from the National Survey of Families and Households in the U.S., found that husbands generally participate in domestic tasks only when both partners hold egalitarian views; minimal household work is undertaken by husbands unless both spouses endorse nontraditional roles. Furthermore, our findings are consistent with previous studies indicating that the relationship between marital quality and cardiovascular health is stronger for women than for men^9,10^.

Relatedly, gender scholarship on hegemonic masculinity suggests that men’s health-related behaviors and engagement with care are shaped by gendered privileges, authority, and norms of self-reliance within heterosexual partnerships^44^. In such contexts, masculine identity pressures may discourage help-seeking and preventive health behaviors even when cardiovascular risk is elevated. Consistent with this idea, recent evidence indicates that sociocultural pressures to convey male gender identity can be associated with lower diagnosis and treatment of common cardiometabolic conditions among men^45^. Accordingly, men’s gender-related vulnerability may be expressed in screening, diagnosis, and treatment uptake—domains not observed in our biomarker-based outcomes—even when biomarker associations with discordance are null. In our setting of older rural couples, discordance may be less likely to increase husbands’ day-to-day role strain under long-established patriarchal arrangements, which may help interpret the null biomarker associations observed among men. Future research should therefore examine whether men become vulnerable in contexts where discordance challenges hegemonic masculinity—manifesting, for example, in substantive renegotiations of men’s responsibilities or perceived authority (e.g., caregiving demands, household labor, or major financial decision-making)—and whether such contexts shape men’s CVD risk management (e.g., screening, diagnosis, treatment uptake), using longitudinal or intervention designs to capture these dynamics.

Building on this gender-relational interpretation, if discordance functions as a chronic relational strain for wives, several psychophysiological and behavioral pathways could plausibly connect that strain to CVD risk biomarkers. Discordance can create negative emotions and marital strain, potentially raising stress hormone levels and increasing cardiovascular risk^9,12^. Additionally, attitudinal discordance may contribute to stress-induced unhealthy lifestyle choices, such as poor dietary habits, which are linked to cardiovascular risk^46^.

Another possible pathway linking attitudinal discordance to cardiovascular risk is through its effect on a sense of purpose in life, a key component of psychological well-being. Sense of purpose is known to decline with age, but social factors like education, income, and marital status can moderate this trend^47^. As individuals age and retire, their social networks tend to shrink, focusing more on close relationships, with the spouse often becoming the primary source of purpose and meaning in later life^48^. Strong discordance in gender role attitudes with one’s spouse could erode this sense of purpose. Evidence from the Health and Retirement Study suggests that negative marital relationships reduce a sense of purpose in life among older adults^47,49^. A lower sense of purpose is associated with an increased risk of depression^49,50^, while a higher sense of purpose is linked to favorable cardiovascular biomarkers (e.g., lower inflammation, higher HDL-C), reduced cardiovascular events (e.g., myocardial infarction, sudden cardiac death, stroke), and lower allostatic load^51–55^. Moreover, individuals with a strong sense of purpose tend to recover emotionally from negative experiences more effectively and perceive stressors as less challenging, which implies that purpose in life is closely tied to healthy coping mechanisms when facing stress^56^.

The discordance in gender role attitudes may contribute to a reduced or unstable sense of purpose in life^57^, which might not be fully captured by the marital quality survey items used in this study. In summary, older adults may be more vulnerable to CVD due to decreased immune function, increased frailty, and the cumulative effects of negative physiological processes and health behaviors^9^, which can result from dysfunctional couple dynamics, including strong discordance in gender role attitudes, emotional strain, and diminished sense of purpose. This may also help explain why spouses with a neutral gender role attitude maintain low levels of SBP and TG: HDL-C ratio regardless of the discordance gap: for these individuals, gender roles may not be central to their sense of life purpose and therefore may not induce significant tension or stress.

It is also noteworthy that not all biomarkers are related to discordance; hsCRP is not associated with attitudinal discordance. Previous studies have shown mixed results regarding the association between marital quality and CRP levels^9,10,58^, making this relationship somewhat inconclusive. There are at least two potential hypotheses that might explain this inconsistency. First, it is possible that the link between marital quality and inflammation is not direct but mediated by other factors, such as changes in diet or sleep^59^. Second, the influence of discordant attitudes on cardiovascular health may be transmitted not through CRP but through other inflammation-related markers, such as interleukin-6^10^ or E-selectin^32^. Additionally, DBP is not associated with attitudinal discordance. This may be due to the characteristics of our older adult sample, as DBP tends to decline with age^60–62^. As DBP decreases in older adulthood, variations in DBP among older adults may become less pronounced, potentially making it more difficult to detect associations in statistical analyses. Together, these null results suggest that discordance may be more strongly linked to hemodynamic and lipid-related pathways than to systemic inflammation as captured by hsCRP.

Because gender role attitudes and CVD risk biomarkers were assessed at the same time point, we cannot establish temporal ordering. The observed association may therefore be bidirectional: attitudinal discordance may contribute to stress-related physiological risk, but poorer cardiovascular health (or health-related functional limitations) may also reshape couples’ role negotiations and perceived discordance. For instance, a wife with a high risk of CVD might experience functional limitations in daily life, which could prompt shifts in her own or her husband’s gender role attitudes, thereby increasing perceived discordance within the couple. Although the Korean Social Life, Health, and Aging Project (KSHAP) is a longitudinal study spanning over a decade, there are currently insufficient waves of data on gender role attitudes and CVD biomarkers to fully investigate causal direction. Fortunately, KSHAP is currently collecting additional longitudinal data while maintaining questions on gender role attitudes, which will allow us to examine causality more rigorously in the future. For the present study, we addressed potential reverse causation by excluding older adults with functional disabilities from the APIM analyses. As shown in Table S4, the results remain consistent, suggesting that it is unlikely that worsening physical health alone explains the discordance in gender role attitudes. Future research should leverage longitudinal couple data with repeated measures of both spouses’ gender role attitudes and CVD biomarkers to establish temporal sequencing and test causal pathways.

This study has limitations related to the measurement of gender role attitudes. Although we used a widely adopted ISSP item capturing views on the gendered division of paid work and household labor, gender ideology is multidimensional and cannot be fully represented by a single statement. A single-item measure also prevents formal reliability assessment and may introduce measurement error, which could attenuate estimated associations—particularly for discordance measures that depend on the difference between spouses’ scores. Future studies would benefit from multi-item scales covering multiple domains (e.g., work–family roles, decision-making authority, childcare, and caregiving) and from examining whether discordance across specific domains shows distinct health implications.

Also, cardiovascular risk biomarkers are shaped not only by psychosocial stressors but also by external and contextual factors that we could not fully capture in our data. In particular, access to and utilization of healthcare services, diet and food environments, and patterns of physical activity may vary across households and rural settings and could influence both cardiometabolic biomarkers and couples’ day-to-day role arrangements. Although we adjusted for several individual-level socioeconomic and behavioral covariates, unmeasured contextual exposures could contribute to residual confounding or to modify the association between attitudinal discordance and cardiovascular risk. Future research would benefit from incorporating more detailed measures of healthcare access and use, diet quality, and objectively measured physical activity, as well as community-level indicators of service availability, to clarify the robustness and boundary conditions of these associations.

Additionally, generalizability remains a concern. Our sample comprises older married couples from two rural South Korean communities, and the magnitude—or even the direction—of associations may differ in urban settings, younger cohorts, or societies with different gender regimes and marital expectations. Future research should therefore replicate these findings in samples with diverse age groups and cultural or social contexts to enhance the external validity of our results. Nonetheless, it is worth noting that discordance in gender role attitudes has been linked to marital quality, and marital quality, in turn, has been associated with cardiovascular risk in various studies conducted in the United States^9,21,22,63,64^. Our findings may also have implications for countries and communities experiencing shifts from traditional to egalitarian gender role attitudes, as rising attitudinal dissimilarity between spouses could have similar health implications^65^.

As a robustness check to examine the consistency of our results, we exclude participants using medication for hypertension or hyperlipidemia from the APIM models. Despite this exclusion, the main results remain consistent. As shown in Table S5, the associations between dissimilarity in spouses’ gender role attitudes and both SBP and log(TG: HDL-C) in women remain statistically significant. Further, we also test if additional marital-level factors—marriage duration and parental status (having any son and any daughter)—account for the observed associations. As summarized in Table S6, including these marital-level covariates does not meaningfully change the main results. This likely reflects the characteristics of the analytic sample: most couples are older and have been married for a long period (mean = 45.6 years), and the vast majority report having at least one son (94.5%) or one daughter (80.5%), limiting within-sample variation in these factors. Nevertheless, other marital-level characteristics not observed in our data may still matter, and results may differ in couple-matched datasets with different demographic or marital profiles.

Our study has several important implications. First, the findings contribute to the ongoing discussion regarding the limited and contradictory evidence linking social networks to the onset of CVD. While previous studies suggest that social networks and support play an essential role in stroke recovery^66^, empirical evidence regarding the association between social networks and CVD onset has been inconclusive. By identifying that discordance in gender role attitudes among married couples can elevate CVD risk markers, this study highlights the significance of couple-level dynamics among older adults as a potential risk factor for CVD onset. Furthermore, we demonstrate that the association between attitudinal discrepancies in spouses and CVD risk markers persists even after controlling for marital quality as perceived by each individual spouse. This finding underscores that, beyond each spouse’s individual attitudes, the interaction between husbands’ and wives’ attitudes can serve as a distinct risk factor associated with CVD risk. Future studies could extend this perspective by examining, for example, whether discordance in parenting attitudes among young or middle-aged couples raising children also elevates CVD risk markers.

Second, our findings call for special attention to the most disadvantaged groups: traditional women with egalitarian husbands and egalitarian women with traditional husbands. Results from marginal effects at the mean show that egalitarian women with traditional husbands have an SBP of 140.90 mmHg and a TG: HDL-C ratio of 3.49, while traditional women with traditional husbands have an SBP of 132.89 mmHg and a TG: HDL-C ratio of 2.07. Similarly, traditional women with egalitarian husbands have an SBP of 139.36 mmHg and a TG: HDL-C ratio of 3.31, whereas egalitarian women with egalitarian husbands have an SBP of 133.88 mmHg and a TG: HDL-C ratio of 1.99.

These results suggest that attitudinal discordance may help identify older wives who warrant closer cardiometabolic risk monitoring and support. In practice, one feasible direction is to integrate brief, spouse-inclusive counseling or education modules into routine community-based care—focusing on modifiable pathways such as stress management, sleep, diet, and physical activity, and, where relevant, couple communication around role expectations and support. Recent community health center trials among older adults with chronic disease illustrate the practicality of delivering structured, spouse-involving behavioral programs in real-world primary care settings, providing a potential implementation template for couple-oriented approaches in later life^67^. Future research should test whether such spouse-inclusive strategies are acceptable and effective for reducing cardiometabolic risk among discordant couples, ideally using longitudinal designs or pragmatic trials and examining whether reductions in perceived strain and improvements in health behaviors mediate biomarker changes. Where discordance reflects entrenched gendered expectations, complementary components that explicitly address gender norms may also be warranted; for example, programs that encourage men to reflect on masculinity and power relations have been used to shift harmful norms^44^. Together with routine cardiovascular screening and stress-management counselling, such components may help couples negotiate role expectations and reduce discordance-related strain.

## Supplementary Information

Below is the link to the electronic supplementary material.

Supplementary Material 1

## Data Availability

All data files are available from the figshare database (https://doi.org/10.6084/m9.figshare.30814394).

## References

[1] WHO. *Cardiovascular diseases (CVDs) key facts* (2021). https://www.who.int/news-room/fact-sheets/detail/cardiovascular-diseases-%28cvds%29

[2] Tsao, C. W. et al. Heart disease and stroke statistics—2023 update: a report from the American Heart Association. *Circulation* **147**, e93–e621. 10.1161/CIR.0000000000001123 (2023). 36695182 10.1161/CIR.0000000000001123 PMC12135016

[3] Korea, S. Causes of Death Statistics, https://kostat.go.kr/statDesc.es?act=view&mid=a10501010000&sttr_cd=S004001 (2023)

[4] Barrett, A. E. Marital trajectories and mental health. *J. Health Soc. Behav.* **41**, 451–464 (2000). 11198568

[5] Lillard, L. A. & Panis, C. W. Marital status and mortality: the role of health. *Demography* **33**, 313–327 (1996). 8875065

[6] Zhang, Z. & Hayward, M. D. Gender, the marital life course, and cardiovascular disease in late midlife. *J. Marriage Fam.* **68**, 639–657 (2006).

[7] Coyne, J. C. et al. Prognostic importance of marital quality for survival of congestive heart failure. *Am. J. Cardiol.* **88**, 526–529 (2001). 11524062 10.1016/s0002-9149(01)01731-3

[8] Gallo, L. C., Troxel, W. M., Matthews, K. A. & Kuller, L. H. Marital status and quality in middle-aged women: associations with levels and trajectories of cardiovascular risk factors. *Health Psychol.* **22**, 453 (2003). 14570528 10.1037/0278-6133.22.5.453

[9] Liu, H. & Waite, L. Bad marriage, broken heart? Age and gender differences in the link between marital quality and cardiovascular risks among older adults. *J. Health Soc. Behav.* **55**, 403–423 (2014). 25413802 10.1177/0022146514556893 PMC4325990

[10] Donoho, C. J., Crimmins, E. M. & Seeman, T. E. Marital quality, gender, and markers of inflammation in the MIDUS cohort. *J. Marriage Fam.* **75**, 127–141 (2013). 24700968 10.1111/j.1741-3737.2012.01023.x PMC3971932

[11] Holt-Lunstad, J., Uchino, B. N., Smith, T. W., Olson-Cerny, C. & Nealey-Moore, J. B. Social relationships and ambulatory blood pressure: structural and qualitative predictors of cardiovascular function during everyday social interactions. *Health Psychol.* **22**, 388 (2003). 12940395 10.1037/0278-6133.22.4.388

[12] Kiecolt-Glaser, J. K. & Newton, T. L. Marriage and health: his and hers. *Psychol. Bull.* **127**, 472 (2001). 11439708 10.1037/0033-2909.127.4.472

[13] Uchino, B. N., Smith, T. W. & Berg, C. A. Spousal relationship quality and cardiovascular risk: dyadic perceptions of relationship ambivalence are associated with coronary-artery calcification. *Psychol. Sci.* **25**, 1037–1042 (2014). 24501110 10.1177/0956797613520015 PMC3984367

[14] Rusbult, C. E. & Van Lange, P. A. Why we need interdependence theory. *Soc. Pers. Psychol. Compass*. **2**, 2049–2070 (2008).

[15] Wang, S. & Li, L. Z. Double jeopardy: the roles of job autonomy and spousal gender ideology in employed women’s mental health. *Appl. Res. Qual. Life*. **18**, 473–490 (2023). 35966806 10.1007/s11482-022-10090-8 PMC9361897

[16] Connell, R. Gender, health and theory: conceptualizing the issue, in local and world perspective. *Soc. Sci. Med.* **74**, 1675–1683 (2012). 21764489 10.1016/j.socscimed.2011.06.006

[17] Bottorff, J. L., Oliffe, J. L., Robinson, C. A. & Carey, J. Gender relations and health research: a review of current practices. *Int. J. Equity Health*. **10**, 60 (2011). 22151578 10.1186/1475-9276-10-60 PMC3293073

[18] Hammarström, A. & Hensing, G. How gender theories are used in contemporary public health research. *Int. J. Equity Health*. **17**, 34 (2018). 29554916 10.1186/s12939-017-0712-x PMC5859645

[19] Regitz-Zagrosek, V. & Gebhard, C. Gender medicine: effects of sex and gender on cardiovascular disease manifestation and outcomes. *Nat. Reviews Cardiol.* **20**, 236–247 (2023). 36316574 10.1038/s41569-022-00797-4 PMC9628527

[20] Davis, S. N. & Greenstein, T. N. Gender ideology: components, predictors, and consequences. *Ann. Rev. Sociol.* **35**, 87–105 (2009).

[21] Cheung, A. K. L. & Choi, S. Y. P. Non-traditional wives with traditional husbands: gender ideology and husband-to-wife physical violence in Chinese society. *Violence Against Women*. **22**, 1704–1724 (2016). 26944714 10.1177/1077801216632615

[22] Lucier-Greer, M. J. *Gender role attitudes: An examination of within-individual malleability and the value of dyadic congruence* (Auburn University, 2012).

[23] Ogolsky, B. G., Dennison, R. P. & Monk, J. K. The role of couple discrepancies in cognitive and behavioral egalitarianism in marital quality. *Sex. Roles* **70**, 329–342. 10.1007/s11199-014-0365-9 (2014).

[24] Gaunt, R. Couple similarity and marital satisfaction: Are similar spouses happier? *J. Pers.* **74**, 1401–1420 (2006). 16958707 10.1111/j.1467-6494.2006.00414.x

[25] Moore, S. M., Uchino, B. N., Baucom, B. R., Behrends, A. A. & Sanbonmatsu, D. Attitude similarity and familiarity and their links to mental health: an examination of potential interpersonal mediators. *J. Soc. Psychol.* **157**, 77–85 (2017). 27065059 10.1080/00224545.2016.1176551 PMC5554447

[26] Baek, J. et al. A prospective sociocentric study of 2 entire traditional korean villages: the Korean Social Life, Health, and Aging Project (KSHAP). *Am. J. Epidemiol.* **193**, 241–255 (2024). 37759338 10.1093/aje/kwad190

[27] Lee, J. M. et al. The Korean social life, health and aging project-health examination cohort. *Epidemiol. Health* **36**, e2014003 (2014). 10.4178/epih/e2014003 PMC4030701 24876995

[28] Youm, Y. et al. Social network properties and self-rated health in later life: comparisons from the Korean social life, health, and aging project and the national social life, health and aging project. *BMC Geriatr.* **14**, 1–15 (2014). 25217892 10.1186/1471-2318-14-102 PMC4236545

[29] Izzo, J. L. & Black, H. R. *Hypertension primer: the essentials of high blood pressure* 3 edn (Lippincott Williams & Wilkins, 2003).

[30] Collaboration, A. P. C. S. A comparison of lipid variables as predictors of cardiovascular disease in the Asia Pacific region. *Ann. Epidemiol.* **15**, 405–413 (2005). 15840555 10.1016/j.annepidem.2005.01.005

[31] Pearson T. A. et al. Markers of inflammation and cardiovascular disease: application to clinical and public health practice: a statement for healthcare professionals from the Centers for Disease Control and Prevention and the American Heart Association. *circulation* **107**, 499–511 (2003). 12551878 10.1161/01.cir.0000052939.59093.45

[32] Yang, Y. C., Schorpp, K. & Harris, K. M. Social support, social strain and inflammation: evidence from a national longitudinal study of US adults. *Soc. Sci. Med.* **107**, 124–135 (2014). 24607674 10.1016/j.socscimed.2014.02.013 PMC4028709

[33] Berry, J. W., Segall, M. H. & Kagitçibasi, Ç. *Handbook of cross-cultural psychology: Social behavior and applications* (Allyn and Bacon, 1997).

[34] McHugh, M. C. & Frieze, I. H. The measurement of gender-role attitudes: a review and commentary. *Psychol. Women Q.* **21**, 1–16 (1997).

[35] Group, I. R. (GESIS Data Archive, Cologne. ZA3880 Data file Version 1.1.0, (2013). 10.4232/1.11564.

[36] Park, A., Bryson, C., Clery, E., Curtice, J. & Phillips, M. *British Social Attitudes: the 30th Report* (NatCen Social Research, 2013).

[37] Radloff, L. S. The CES-D scale: a self-report depression scale for research in the general population. *Appl. Psychol. Meas.* **1**, 385–401 (1977).

[38] Carr, D., Cornman, J. C. & Freedman, V. A. Marital quality and negative experienced well-being: an assessment of actor and partner effects among older married persons. *J. Gerontol. Ser. B Psychol. Sci. Soc. Sci.* **71**, 177–187 (2016). 26329115 10.1093/geronb/gbv073 PMC4701126

[39] Carr, D., Freedman, V. A., Cornman, J. C. & Schwarz, N. Happy marriage, happy life? Marital quality and subjective well-being in later life. *J. Marriage Fam.* **76**, 930–948 (2014). 25221351 10.1111/jomf.12133 PMC4158846

[40] Cook, W. L. & Kenny, D. A. The actor–partner interdependence model: a model of bidirectional effects in developmental studies. *Int. J. Behav. Dev.* **29**, 101–109 (2005).

[41] Knuiman, M. W., Divitini, M. L., Bartholomew, H. C. & Welborn, T. A. Spouse correlations in cardiovascular risk factors and the effect of marriage duration. *Am. J. Epidemiol.* **143**, 48–53 (1996). 8533746 10.1093/oxfordjournals.aje.a008656

[42] Ross, C. E. The division of labor at home. *Soc. Forces*. **65**, 816–833 (1987).

[43] Greenstein, T. N. Husbands’ participation in domestic labor: Interactive effects of wives’ and husbands’ gender ideologies. *J. Marriage Fam.* **58**, 585–595 (1996).

[44] Jewkes, R. et al. Hegemonic masculinity: combining theory and practice in gender interventions. *Cult. Health Sex.* **17**, 112–127 (2015). 10.1080/13691058.2015.1085094 PMC4706037 26680535

[45] Glasser, N. J. et al. Male gender expressivity and diagnosis and treatment of cardiovascular disease risks in men. *JAMA Netw. Open.* **7**, e2441281–e2441281 (2024). 39453653 10.1001/jamanetworkopen.2024.41281 PMC11512345

[46] Robles, T. F. & Kiecolt-Glaser, J. K. The physiology of marriage: pathways to health. *Physiol. Behav.* **79**, 409–416 (2003). 12954435 10.1016/s0031-9384(03)00160-4

[47] Hill, P. L. & Weston, S. J. Evaluating eight-year trajectories for sense of purpose in the health and retirement study. *Aging Ment. Health*. **23**, 233–237 (2019). 29212348 10.1080/13607863.2017.1399344

[48] Carstensen, L. L., Fung, H. H. & Charles, S. T. Socioemotional selectivity theory and the regulation of emotion in the second half of life. *Mot. Emot.* **27**, 103–123 (2003).

[49] Irani, E., Park, S. & Hickman, R. L. Negative marital interaction, purpose in life, and depressive symptoms among middle-aged and older couples: evidence from the Health and Retirement Study. *Aging Ment. Health*. **26**, 860–869 (2022). 33769159 10.1080/13607863.2021.1904831 PMC8742630

[50] Wood, A. M. & Joseph, S. The absence of positive psychological (eudemonic) well-being as a risk factor for depression: a ten year cohort study. *J. Affect. Disord.* **122**, 213–217 (2010). 19706357 10.1016/j.jad.2009.06.032

[51] Cohen, R., Bavishi, C. & Rozanski, A. Purpose in life and its relationship to all-cause mortality and cardiovascular events: a meta-analysis. *Psychosom. Med.* **78**, 122–133 (2016). 26630073 10.1097/PSY.0000000000000274

[52] Kim, E. S., Chen, Y., Nakamura, J. S., Ryff, C. D. & VanderWeele, T. J. Sense of purpose in life and subsequent physical, behavioral, and psychosocial health: an outcome-wide approach. *Am. J. Health Promot.* **36**, 137–147 (2022). 34405718 10.1177/08901171211038545 PMC8669210

[53] Lee, S. H. et al. Psychological well-being and salivary markers of inflammation: the moderating effect of age. *Appl. Psychol, Health Well Being*. **15**, 466–478 (2023). 35851762 10.1111/aphw.12389

[54] Steptoe, A. & Fancourt, D. Leading a meaningful life at older ages and its relationship with social engagement, prosperity, health, biology, and time use. *Proc. Natl. Acad. Sci.* **116**, 1207–1212 (2019). 30617082 10.1073/pnas.1814723116 PMC6347683

[55] Zilioli, S., Slatcher, R. B., Ong, A. D. & Gruenewald, T. L. Purpose in life predicts allostatic load ten years later. *J. Psychosom. Res.* **79**, 451–457 (2015). 26526322 10.1016/j.jpsychores.2015.09.013 PMC4684637

[56] Kim, E. S., Delaney, S. W. & Kubzansky, L. D. Sense of purpose in life and cardiovascular disease: underlying mechanisms and future directions. *Curr. Cardiol. Rep.* **21**, 1–11 (2019). 31673815 10.1007/s11886-019-1222-9 PMC10683927

[57] Deutsch, F. M. & Saxon, S. E. Traditional ideologies, nontraditional lives. *Sex. Roles* **38**, 331–362 (1998).

[58] Salinger, J. M. & Whisman, M. A. Marital dissolution, marital discord, and C-reactive protein: results from the Irish longitudinal study on ageing. *Health Psychol.* **40**, 459 (2021). 34435797 10.1037/hea0001083

[59] Kiecolt-Glaser, J. K. & Wilson, S. J. Lovesick: how couples’ relationships influence health. *Ann. Rev. Clin. Psychol.* **13**, 421–443 (2017). 28301763 10.1146/annurev-clinpsy-032816-045111 PMC5549103

[60] Burt, V. L. et al. Prevalence of hypertension in the US adult population: results from the Third National Health and Nutrition Examination Survey, 1988–1991. *Hypertension* **25**, 305–313 (1995). 7875754 10.1161/01.hyp.25.3.305

[61] Franklin, S. S. et al. Hemodynamic patterns of age-related changes in blood pressure: the Framingham Heart Study. *Circulation* **96**, 308–315 (1997). 9236450 10.1161/01.cir.96.1.308

[62] Pinto, E. Blood pressure and ageing. *Postgrad. Med. J.* **83**, 109–114 (2007). 17308214 10.1136/pgmj.2006.048371 PMC2805932

[63] Juni, S. & Grimm, D. W. Marital satisfaction as a function of dyadic gender-role constellations. *Am. J. Fam. Ther.* **22**, 106–112 (1994).

[64] Lye, D. N. & Biblarz, T. J. The effects of attitudes toward family life and gender roles on marital satisfaction. *J. Fam. Issues*. **14**, 157–188 (1993).

[65] Palley, M. L. Women’s status in South Korea: tradition and change. *Asian Surv.* **30**, 1136–1153 (1990).

[66] Berkman, L. F. & Krishna, A. in *Social Epidemiology* eds Lisa F Berkman, Ichiro Kawachi, & M Maria Glymour, Ch. 7, 234–289 (Oxford University Press, 2014).

[67] Yang, C. et al. A couple-based intervention for Chinese older adults with type 2 diabetes: a randomized clinical trial. *JAMA Netw. Open.* **8**, e2452168 (2025). 39745703 10.1001/jamanetworkopen.2024.52168 PMC11696449

