# Supplementary material for: Discordance in gender role attitudes between spouses and its relationship with the risk biomarkers of cardiovascular diseases: a couple-level analysis
Source: Sci Rep. 2026 Mar 3;16:11881. doi: 10.1038/s41598-026-41697-8 (PMC13066615; doi:10.1038/s41598-026-41697-8)
Supplement: Supplementary file 1 — Supplementary Material 1 [file 41598_2026_41697_MOESM1_ESM.docx]

**Supplementary Material**

**Table S1. Factor loadings for marital quality (*N* = 616 individuals)**

| **Item** | **Positive marital quality** | **Negative marital quality** |
| --- | --- | --- |
| How close do you feel is your relationship with spouse? | 0.33 | -0.05 |
| Do you and spouse spend free time together or apart? | 0.30 | 0.04 |
| How often can you open up to spouse? | 0.61 | 0.10 |
| How often can you rely on spouse? | 0.63 | 0.14 |
| How often does spouse make too many demands on you? | -0.11 | 0.43 |
| How often does spouse criticize you? | -0.20 | 0.45 |

**Table S2. Distribution of gender role attitudes in women and men (*N* = 308 couples)**

|  | **Wife's gender role attitude** | | | | | **Total** |
| --- | --- | --- | --- | --- | --- | --- |
| **Husband's gender role attitude** | **Very traditional** | **Traditional** | **Neutral** | **Egalitarian** | **Very egalitarian** |  |
| **Very traditional** | 12 (3.9) | 13 (4.2) | 10 (3.3) | 13 (4.2) | 7 (2.3) | 55 (17.9) |
| **Traditional** | 8 (2.6) | 24 (7.8) | 11 (3.6) | 28 (9.1) | 10 (3.3) | 81 (26.3) |
| **Neutral** | 6 (2.0) | 16 (5.2) | 12 (3.9) | 24 (7.8) | 7 (2.3) | 65 (21.1) |
| **Egalitarian** | 8 (2.6) | 17 (5.5) | 12 (3.9) | 38 (12.3) | 13 (4.2) | 88 (28.6) |
| **Very egalitarian** | 3 (1.0) | 3 (1.0) | 2 (0.7) | 9 (2.9) | 2 (0.7) | 19 (6.2) |
| **Total** | 37 (12.0) | 73 (23.7) | 47 (15.3) | 112 (36.4) | 39 (12.7) | 308 (100.0) |

*Note*. Values in each cell indicate n (cell %).

**Table S3. Correlation between spouses’ risk markers of cardiovascular disease (*N* = 308 couples)**

| **Variables** | **Correlation coefficient (Pearson's r or Spearman's ρ)** |
| --- | --- |
| SBP (mmHg) | 0.04 |
| DBP (mmHg) | 0.13* |
| hsCRP (mg/L) | 0.03 |
| TG (mg/dL) | 0.19* |
| HDL-C (mg/dL) | 0.04 |
| TG:HDL-C ratio | 0.14* |

*Note*. SBP = systolic blood pressure; DBP = diastolic blood pressure; hsCRP = high-sensitivity C-reactive protein; TG = triglyceride; HDL-C = high-density lipoprotein cholesterol. Considering high skewness of hsCRP, TG, and TG:HDL-C ratio, Spearman's ρ is measured. Pearson's r is measured for SBP, DBP, and HDL-C.

**p* < .05.

**Table S4. Actor-partner interdependence models (APIMs) after excluding respondents with functional disability ^a^**

|  | **Women** | | **Men** | |
| --- | --- | --- | --- | --- |
|  | **Model A** | **Model B** | **Model A** | **Model B** |
|  | Coefficient [95% CI] | Coefficient [95% CI] | Coefficient [95% CI] | Coefficient [95% CI] |
| **DV: SBP** |  |  |  |  |
| Wife attitude | 0.89 [-0.43, 2.21] | 3.28* [0.35, 6.21] | 0.81 [-0.60, 2.21] | 0.70 [-2.49, 3.90] |
| Husband attitude | -0.19 [-1.48, 1.11] | 2.51 [-0.72, 5.74] | -0.73 [-2.16, 0.71] | -0.84 [-4.37, 2.69] |
| Wife × Husband attitude |  | -0.88+ [-1.84, 0.09] |  | 0.04 [-1.02, 1.09] |
| Observation | 291 | | | |
| **DV: DBP** |  |  |  |  |
| Wife attitude | 0.27 [-0.49, 1.03] | 0.21 [-1.50, 1.91] | -0.73+ [-1.48, 0.03] | -0.77 [-2.48, 0.94] |
| Husband attitude | -0.13 [-0.88, 0.62] | -0.21 [-2.09, 1.67] | 0.032 [-0.73, 0.80] | -0.02 [-1.91, 1.87] |
| Wife × Husband attitude |  | 0.025 [-0.54, 0.59] |  | 0.02 [-0.55, 0.58] |
| Observation | 291 | | | |
| **DV: log(hsCRP)** |  |  |  |  |
| Wife attitude | 0.04 [-0.06, 0.14] | 0.13 [-0.12, 0.38] | 0.01 [-0.08, 0.10] | 0.14 [-0.09, 0.37] |
| Husband attitude | -0.09+ [-0.19, 0.01] | -0.01 [-0.23, 0.21] | 0.02 [-0.07, 0.11] | 0.14 [-0.07, 0.35] |
| Wife × Husband attitude |  | -0.03 [-0.10, 0.04] |  | -0.04 [-0.11, 0.03] |
| Observation | 273 | | | |
| **DV: log(TG:HDL-C ratio)** |  |  |  |  |
| Wife attitude | 0.03 [-0.03, 0.08] | 0.19** [0.07, 0.31] | -0.04 [-0.10, 0.01] | -0.01 [-0.14, 0.12] |
| Husband attitude | -0.02 [-0.08, 0.03] | 0.16* [0.03, 0.30] | -0.02 [-0.08, 0.04] | 0.02 [-0.12, 0.15] |
| Wife × Husband attitude |  | -0.06** [-0.10, -0.02] |  | -0.01 [-0.05, 0.03] |
| Observation | 291 | | | |

*Note*. In all models, we control for age, education, household income, working status, township, drinking, smoking, obesity, medication uses for hypertension and hyperlipidemia, depressive symptoms, and positive and negative marital quality. DV = dependent variable; CI = confidence interval; SBP = systolic blood pressure; DBP = diastolic blood pressure; TG = triglyceride; HDL-C = high-density lipoprotein cholesterol.

^a^ Respondents with either K-ADL > 18 or K-IADL > 1 were excluded (n = 17 couples). K-ADL = Korean version of activities of daily living; K-IADL = Korean version of instrumental activities of daily living.

+*p* < .10; **p* < .05; ***p* < .01; ****p* < .001.

**Table S5. Actor-partner interdependence models (APIMs) after excluding medication users**

|  | **Women** | | **Men** | |
| --- | --- | --- | --- | --- |
|  | **Model A** | **Model B** | **Model A** | **Model B** |
|  | Coefficient [95% CI] | Coefficient [95% CI] | Coefficient [95% CI] | Coefficient [95% CI] |
| **DV: SBP** |  |  |  |  |
| Wife attitude | 1.15 [-0.93, 3.23] | 4.95* [0.64, 9.25] | 0.49 [-2.00, 2.98] | -1.46 [-6.85, 3.93] |
| Husband attitude | -0.63 [-2.61, 1.34] | 3.78 [-1.03, 8.59] | -2.35+ [-4.87, 0.17] | -4.65 [-10.85, 1.55] |
| Wife × Husband attitude |  | -1.42* [-2.83, -0.00] |  | 0.72 [-1.06, 2.49] |
| Observation | 95 | | | |
| **DV: DBP** |  |  |  |  |
| Wife attitude | 0.55 [-0.83, 1.93] | 0.89 [-2.03, 3.82] | -0.83 [-2.20, 0.53] | -1.58 [-4.55, 1.39] |
| Husband attitude | -0.92 [-2.23, 0.39] | -0.53 [-3.80, 2.74] | -0.93 [-2.31, 0.46] | -1.82 [-5.23, 1.59] |
| Wife × Husband attitude |  | -0.13 [-1.09, 0.83] |  | 0.28 [-0.70, 1.26] |
| Observation | 95 | | | |
| **DV: log(TG:HDL-C ratio)** |  |  |  |  |
| Wife attitude | 0.03 [-0.03, 0.09] | 0.20** [0.07,0.33] | -0.05 [-0.11, 0.01] | -0.05 [-0.18, 0.08] |
| Husband attitude | -0.02 [-0.08, 0.04] | 0.18* [0.03, 0.32] | -0.04 [-0.10, 0.02] | -0.04 [-0.19, 0.10] |
| Wife × Husband attitude |  | -0.07** [-0.11, -0.02] |  | 0.00 [-0.04, 0.05] |
| Observation | 237 | | | |

*Note*. In all models, we control for age, education, household income, working status, township, drinking, smoking, obesity, depressive symptoms, and positive and negative marital quality. We control for medication uses for hyperlipidemia when DV is SBP or DBP, and control for medication uses for hypertension when DV is log(TG:HDL-C ratio). DV = dependent variable; CI = confidence interval; SBP = systolic blood pressure; DBP = diastolic blood pressure; TG = triglyceride; HDL-C = high-density lipoprotein cholesterol.

+*p* < .10; **p* < .05; ***p* < .01; ****p* < .001.

**Table S6. Actor-partner interdependence models (APIMs) with additional marital-level factors**

|  | **Women** | | **Men** | |
| --- | --- | --- | --- | --- |
|  | **Model A** | **Model B** | **Model A** | **Model B** |
|  | Coefficient [95% CI] | Coefficient [95% CI] | Coefficient [95% CI] | Coefficient [95% CI] |
| **DV: SBP** |  |  |  |  |
| Wife attitude | 0.66 [0.64, 1.97] | 2.98* [0.07, 5.89] | 0.72 [0.64, 2.08] | 1.20 [1.87, 4.27] |
| Husband attitude | -0.08 [1.36, 1.19] | 2.57 [-0.67, 5.81] | -0.66 [2.03, 0.71] | -0.11 [3.55, 3.33] |
| Wife × Husband attitude |  | -0.86+ [1.83, 0.11] |  | -0.18 [1.20, 0.85] |
| Marriage duration (years) | 0.1 [0.17, 0.36] | 0.07 [0.20, 0.34] | -0.19 [0.44, 0.06] | -0.20 [0.45, 0.05] |
| Any son | -0.26 [7.03, 6.51] | -0.54 [7.28, 6.20] | 2.12 [5.05, 9.28] | 2.09 [5.08, 9.25] |
| Any daughter | -0.68 [4.52, 3.16] | -0.96 [4.79, 2.87] | -3.35 [7.39, 0.68] | -3.41+ [7.46, 0.63] |
| Observation | 308 | | | |
| **DV: DBP** |  |  |  |  |
| Wife attitude | 0.27 [0.48, 1.02] | -0.09 [1.77, 1.59] | -0.53 [1.27, 0.20] | -0.29 [1.95, 1.37] |
| Husband attitude | 0.13 [0.60, 0.86] | -0.28 [2.15, 1.59] | 0.04 [0.70, 0.78] | 0.32 [1.54, 2.17] |
| Wife × Husband attitude |  | 0.13 [0.43, 0.70] |  | -0.09 [0.64, 0.46] |
| Marriage duration (years) | 0.04 [0.11, 0.19] | 0.04 [0.11, 0.20] | -0.02 [0.15, 0.12] | -0.02 [0.15, 0.11] |
| Any son | 0.46 [3.43, 4.35] | 0.5 [3.39, 4.39] | 2.1 [1.77, 5.96] | 2.09 [1.78, 5.95] |
| Any daughter | 0.09 [2.11, 2.30] | 0.14 [2.08, 2.35] | -1.29 [3.47, 0.88] | -1.32 [3.51, 0.86] |
| Observation | 308 | | | |
| **DV: log(hsCRP)** |  |  |  |  |
| Wife attitude | -0.09+ [0.19, 0.00] | -0.01 [0.22, 0.20] | 0.00 [0.08, 0.09] | 0.07 [0.14, 0.27] |
| Husband attitude | 0.04 [0.05, 0.13] | 0.13 [0.11, 0.37] | 0.00 [0.09, 0.09] | 0.07 [0.15, 0.30] |
| Wife × Husband attitude |  | -0.03 [0.10, 0.04] |  | -0.02 [0.09, 0.04] |
| Marriage duration (years) | 0.19 [0.30, 0.68] | 0.18 [0.31, 0.68] | 0.01 [0.00, 0.03] | 0.01 [0.01, 0.03] |
| Any son | -0.06 [0.34, 0.22] | -0.06 [0.34, 0.22] | -0.26 [0.73, 0.22] | -0.26 [0.73, 0.22] |
| Any daughter | 0.01 [0.01, 0.03] | 0.01 [0.01, 0.02] | 0.10 [0.17, 0.36] | 0.09 [0.18, 0.36] |
| Observation | 289 | | | |
| **DV: log(TG:HDL-C ratio)** |  |  |  |  |
| Wife attitude | 0.02 [0.03, 0.08] | 0.18**  [0.06, 0.30] | -0.05+ [0.10, 0.01] | 0.01 [0.12, 0.13] |
| Husband attitude | -0.02 [0.07, 0.04] | 0.16*  [0.03, 0.29] | -0.01 [0.07, 0.04] | 0.05 [0.09, 0.19] |
| Wife × Husband attitude |  | -0.06**  [-0.10, -0.02] |  | -0.02 [0.06, 0.02] |
| Marriage duration (years) | 0.01* [0.00, 0.02] | 0.01+  [-0.00, 0.02] | 0.00 [0.01, 0.01] | -0.01 [0.02, 0.00] |
| Any son | 0.22 [-0.06, 0.50] | 0.20 [-0.08, 0.48] | 0.00 [0.29, 0.28] | -0.01 [0.29, 0.28] |
| Any daughter | -0.01 [-0.17, 0.15] | -0.03 [-0.19, 0.13] | -0.10 [0.26, 0.06] | -0.11 [0.27, 0.05] |
| Observation | 308 | | | |

*Note*. In all models, we control for age, education, household income, working status, township, drinking, smoking, obesity, medication uses for hypertension and hyperlipidemia, depressive symptoms, and positive and negative marital quality. DV = dependent variable; SBP = systolic blood pressure; DBP = diastolic blood pressure; hsCRP = high-sensitivity C-reactive protein; TG = triglyceride; HDL-C = high-density lipoprotein cholesterol.

+*p* < .10; **p* < .05; ***p* < .01; ****p* < .001.
